# Analytical strategies for the marble burying test: avoiding impossible predictions and invalid p-values

**DOI:** 10.1186/s13104-015-1062-7

**Published:** 2015-04-11

**Authors:** Stanley E Lazic

**Affiliations:** In Silico Lead Discovery, Novartis Institutes for Biomedical Research, Basel, Switzerland

**Keywords:** Autism, Count data, Generalised linear model, Marble burying test, Poisson, Pseudoreplication, Reproducible research, Valproic acid

## Abstract

**Background:**

The marble burying test is used to measure repetitive and anxiety-related behaviour in rodents. The number of marbles that animals bury are count data (non-negative integers), which are bounded below by zero and above by the number of marbles present. Count data are often analysed using normal linear models, which include the t-test and analysis of variance (ANOVA) as special cases. Linear models assume that the data are unbounded and that the variance is constant across groups. These requirements are rarely met with count data, leading to 95% confidence intervals that include impossible values (less than zero or greater than the number of marbles present), misleading p-values, and impossible predictions. Transforming the data or using nonparametric methods are common alternatives but transformations do not perform well when many zero values are present and nonparametric methods have several drawbacks.

**Findings:**

The problems with using normal linear models to analyse marble burying data are demonstrated and generalised linear models (GLMs) are introduced as more appropriate alternatives.

**Conclusions:**

GLMs have been specifically developed to deal with count and other types of non-Gaussian data, are straightforward to use and interpret, and will lead to more sensible inferences.

**Electronic supplementary material:**

The online version of this article (doi:10.1186/s13104-015-1062-7) contains supplementary material, which is available to authorized users.

## Background

**Figure 1:**
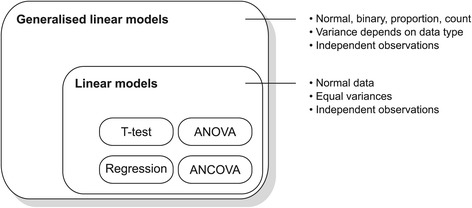
Relationship between statistical models. T-tests, ANOVA, regression, and ANCOVA are all specific examples of linear models and have the same assumptions, including normally distributed data, homogeneity of variance, and independent observations. Generalised linear models include all linear models, but also include models that can handle other types of data. The mean-variance relationship will depend on the specific model. Note that all models assume that observations are independent [41].

The marble burying test is commonly used to quantify anxiety, obsessive-compulsive, or repetitive behaviour in rodents [1-3]. Performance on the marble burying test is also associated with general digging behaviour [4-7], and so the underlying construct being measured is still unclear. Regardless, the typical protocol places 10–25 marbles in a cage containing sawdust or similar material. The marbles are usually arranged in a grid pattern and animals are allowed to explore the cage for a fixed period of time—usually 30 minutes. The main outcome is the total number of marbles buried under the sawdust, but other measures have been used such as the latency to bury the first marble or the amount of time spent burying. The majority of analyses use normal linear models—of which the t-test, ANOVA, and regression are specific examples—to analyse the number of marbles buried (Figure 1). Occasionally, nonparametric methods are employed. Parametric analyses assume that the data (or equivalently, the errors) take a certain distributional form. Choosing an appropriate distribution from the many available is a decision made by the analyst, but invariably the normal or Gaussian distribution is used, likely because neuroscientists are unaware that other options exist.

Data derived from the marble burying test have several properties that make the use of normal linear models questionable. First, the data often contain censored observations. These occur when animals bury all of the marbles present and therefore it is unknown whether the animals would have buried more marbles had they been available. Thus, the relationship between the underlying construct (degree of anxiety/compulsiveness/digging) and the measured outcome (number of marbles buried) breaks down once all the marbles are buried. Censored values can bias estimates of treatment effects and lead to a loss of sensitivity; for example, a treatment would appear ineffective if animals in both the treated and control group bury almost all of the marbles. If more marbles are used, then the control group may bury more than the treated group. Methods exist for analysing censored data but will not be considered here because simply increasing the number of marbles used or reducing the time that animals have to bury them can remove, or greatly reduce, the number of censored observations. Sugimoto and colleagues counted the number of marbles buried at 10, 20, and 30 minutes and showed how differences at early time points between control and treated groups tend to diminish by 30 minutes [8]. At 10 minutes the lowest dose group (1 mg/kg fluvoxamine; Figure one in their paper) buried nearly three times as many marbles as the control group, but by 30 minutes they buried the same number. Thus, an incorrect conclusion would have been reached if the final time point was the only one examined. Santini also examined multiple time points and differences between groups at 30 minutes were also smaller compared with earlier time points (Figure two A in their paper) [9]. The number of marbles and the time the animals have to bury them should be optimised to ensure that the test has good sensitivity and to avoid censored observations.

Second, the number of marbles buried are counts, which means they are non-negative integers (0,1,2,3, …) and therefore have a minimum value of zero. Such data will not follow a normal distribution when the counts are small, but will become increasingly normal as the counts get larger. A normal model assumes that the data can take any real number (−*∞*, +*∞*), and there is nothing to constrain impossible values. This is a problem because 95% confidence interval (CIs) and predicted values can include negative values, which cannot occur with count data. For example, when using a normal model the numbers {0, 0, 0, 0, 1, 2, 5, 9} have a mean of 2.12 and a symmetric 95% CI of -0.14 to 4.39. The negative values indicate that a normal model is unsuitable, and the reference distribution used to calculate p-values will contain impossible values, making the p-values invalid.

The third property that makes a normal model dubious is that in addition to a minimum value of zero, there is also a maximum value—the total number of marbles available, making the data bounded from above as well as below. Since a normal model has no constraints on possible values that the data may take, confidence intervals can include values greater than the total number of marbles available. One solution is to design the experiment so that the number of marbles used and the time animals have to bury them produces results that are not too close to the upper boundary (which will also ensure censored observations are unlikely).

**Figure 2:**
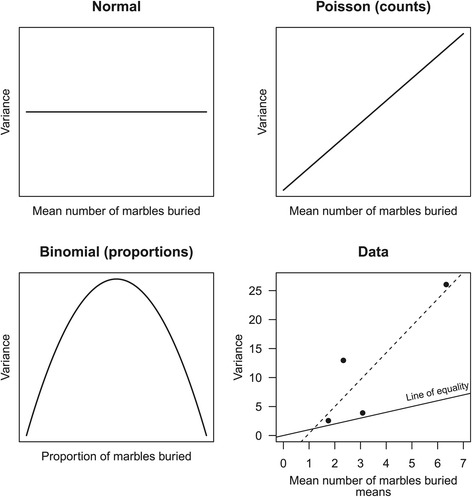
Mean-variance relationships for different models. A normal model assumes that the variance is constant and does not depend on the mean. A Poisson model assumes that the variance is equal to the mean. A binomial model has maximal variance at a proportion of 0.5 and progressively smaller variances on either side. The means and variances for the four experimental groups from Mehta et al. are plotted [18], and if the points fall along the line of equality then a Poisson model would be suitable. The variance is greater than the mean in this data, indicating overdispersion. Dashed line = regression line.

Finally, a normal model assumes that the variance of the data is independent of the mean, which is also referred to as homogeneity of variance or homoskedasticity. With count data however the variance is proportional to the mean—groups with higher means will have higher variances (Figure 2). In addition, since the total number of marbles is bounded, it may happen that at high mean values the variance decreases again. Given the nature of the data obtained from the marble burying test, it is clear that in many cases a normal model will be inappropriate and other options will need to be considered.

One approach to deal with unequal variances and skewness is to transform the data; for example, by taking the square-root or logarithm of the counts. While such transformations often improve the distributional properties of the data, they may not work adequately in all situations, in particular when there are many zeros. Log transformation is not recommended for count data [10], partly because the log of zero is undefined, making it necessary to add a small value to all of the counts.

Another approach often used when the assumptions of a parametric test are not met is to switch to nonparametric methods. Nonparametric tests have a number of undesirable features. First, most nonparametric methods analyse ranks rather than the original values, which results in a loss of information. Second, parameter estimates and their confidence intervals are usually not provided by standard statistical software, only p-values. This means that it is not possible to estimate important values such as a maximum response or the EC _50_ (concentration that gives half of the maximal response) in a dose-response design; the only output is an uninformative “the groups are different (p < 0.05)”. Third, only simple designs can be analysed in a straightforward manner with standard software. Covariates cannot be included (e.g. adjusting for baseline) and it is difficult to include multiple explanatory variables in the analysis and to test for interactions. Some work-arounds can b e employed such as testing groups individually, but then the interpretation is more complex and such analyses tend to be conducted and interpreted inappropriately by neuroscientists [11].

The key message of this paper is that rather than using nonparametric tests or transforming the data to fit a normal model, it is often better to use an appropriate parametric model in the first place. In other words, select a model that fits the data rather than mangle the data to fit a normal model. Generalised linear models (GLMs) were developed by Nelder and Wedderburn in the 1970s and are routinely used in many disciplines [12]. As their name suggests, GLMs are more general versions of normal linear models. GLMs can be used for any standard analysis that assumes normality, equal variances, and unbounded data, but they can also be used for binary (0 or 1), proportion (0 to 1), count (non-negative integers), and positively skewed data. A detailed discussion of GLMs is beyond the scope of this article but can be found in references [13-15]. Hilbe provides an introductory book on the analysis of count data suitable for biologis ts [16], as well as a comprehensive but more technical book [17]. Briefly, GLMs use one of several distributions from the exponential family to describe the data. Examples of distributions from the exponential family include the Gaussian (for normal data), the gamma (for data with a positive skew), the Poisson and negative binomial (for count data), and the binomial (for binary and proportion data). The family is the only option that needs to be specified when analysing the data. Two other features of GLMs include the *link function*, which describes how the mean of the data is related to the explanatory variables and associated parameters, and the *inverse link function*, which restricts the predicted values to lie within allowable bounds, such as non-negative values for count data. Each distribution has a default or “canonical” link that is typically used, and therefore does not need to be specified during the analysis (other link functions are available but they will not be considered here). One difference with interpreting the results of GLMs is that the coefficients are not on the original scale of the data. If interpretation of coefficients is desired, then the coefficients need to be back-transformed onto the original scale by applying the inverse link function. For example, the link function for a Poisson GLM is the log-link, and therefore taking the antilog returns coefficients on the original scale (i.e. in units of marbles). GLMs are implemented in all major statistics packages and have few “barriers-to-entry”. An appropriate family for the data needs to be selected, but then analysis proceeds as usual.

## Methods

Marble burying data from Mehta and colleagues were used, and the experimental details can be found in the original paper [18]; protocols were approved by the University of Pennsylvania Institutional Animal Care and Use Committees and were conducted in accordance with National Institutes of Health guidelines. Briefly, 14 pregnant mice were randomly assigned to receive valproic acid (VPA; *n*=9) or a saline injection (*n*=5). VPA was used to generate autistic-like behaviour in the offspring (*n*_offspring_=48). Half of the offspring in each condition were also randomly given MPEP (2-methyl-6-phenylethyl-pyrididine), a metabotropic glutamate receptor 5 antagonist, or saline injections. MPEP was hypothesised to correct some of the features induced by VPA and the number of marbles buried out of 20 was an outcome. There are three hypotheses of interest: (1) does VPA affect the number of marbles buried, (2) does MPEP affect the number of marbles buried, and (3) does the existence or strength of MPEP’s effect depend on whether animals were exposed to VPA *in utero* (interaction effect).

This data set is more complex than it initially appears because VPA was applied to pregnant females and MPEP to the individual offspring of those females. This is called a split-unit or split-plot design because there are two types of experimental units. An experimental unit is the smallest division of sample material that can be randomly and independently assigned to different treatment conditions. The sample size, or “n”, is the number of experimental units and must be determined correctly to obtain valid p-values. When testing the effect of VPA, the experimental units are the pregnant females (*n*=14); when testing the effect of MPEP, the experimental units are the individual offspring (*n*=48) [19,20]. The power to detect an effect of VPA is lower than for MPEP because of the smaller sample size, and ignoring the split-unit treatment structure during the analysis can give both too many false positives and false negatives [21-24]. Generalised mixed-effects models could be used for such data but are beyond the scope of this article [25]. Rodent studies using split-unit designs are becoming increasingly popular as new disease models have been developed that apply an intervention to pregnant females to induce pathology in the offspring [26], and due to recent interest in the epigenetic transfer of paternal traits [27]. Since the split-unit structure is rarely taken into account, the statistical results from these studies are largely uninterpretable [24,28]. The implications of the split-unit design will generally be ignored to simplify the comparison of methods. The results of a generalised mixed-effects model are however reported, both to compare with the other models and for those readers who are interested in the effects of VPA and MPEP.

The data were analysed using four parametric GLMs and one nonparametric analysis, but this does not exhaust all possibilities. The first analysis was a standard 2-way ANOVA with VPA and MPEP as factors and is a specific example of a normal linear model (and equivalent to a Gaussian GLM with the identity link). This can be thought of as the “standard analysis” that would most commonly be used and which the other analyses are compared against. With this model it is assumed that the data can be reasonably approximated by a normal distribution, can theoretically take any value, the variances are equal in all groups, and the responses of the individual animals are independent of each other. The second model assumes that the data can be described by a Poisson distribution (with the default log link), which is appropriate for count data. One assumption of a Poisson GLM is that the variance is equal to the mean. This assumption needs to be verified, much like the assumption of equal variances with a normal model. Assumed mean-variance relationships for different GLMs are shown in Figure 2, along with the relationship observed in the actual data. With count data, it often happens that the variance is greater than the mean, and is referred to as *overdispersion*. Overdispersion can occur for a variety of reasons, including (1) an important variable has been omitted from the model, (2) an important interaction term was not included, (3) the presence of outliers in the data, or (4) a positive correlation between responses, which might indicate a lack of independence of individual responses [17]. If the data are overdispersed, then a Poisson model will have confidence intervals that are artificially precise and p-values that are too small. An estimate of overdispersion is often provided from the output of a GLM analysis but it can also be calculated by dividing the residual deviance by the residual degrees of freedom (which also should be provided in the output of an analysis), and as a rule-of-thumb, if this value is greater than 1.25 [17] or 1.5 [29], then a Poisson model may be unsuitable. The overdispersion in the Mehta data was estimated to be 2.96, which is well above these thresholds. The Poisson model has provided useful information that cannot be obtained with a standard analysis. Why are the data overdispersed? Was an important variable omitted? This is indeed the case, the overdispersion is the result of litter-effects [24]. Multiple litters were used in this experiment and animals within litters tended to bury a similar number of marbles compared with animals between litters. Overdispersion can be remedied in several ways; if it is due to a missing variable or interaction term in the analysis, then including these will remove the overdispersion, and this should be the first option considered. Another method of dealing with overdispersion is to use a quasi-Poisson GLM, which is the third GLM considered. A quasi-Poisson GLM estimates the amount of overdispersion and scales the standard errors upwards to give the appropriate confidence intervals and p-values; the coefficients are identical to the Poisson model, only the uncertainty in the estimates differ. The fourth GLM is a negative binomial model, which is also used for count data but is more flexible than the Poisson in that the variance can exceed the mean. It is therefore an alternative to a quasi-Poisson model when overdispersion is present. The final analysis uses a nonparametric Wilcoxon rank-sums test. Since it is not possible to calculate an interaction effect, tests between individual groups were conducted, and no correction for multiple testing was used.

The results of the different models are compared in four ways. First, 95% confidence intervals are examined to see whether they include impossible values for parameters. Second, visual predictive checks are used to compare data generated from the model to the original data, to see whether there are any discrepancies [30], pp. 158–163. Third, the goodness-of-fit (GOF) of the models was tested. Fourth, models are compared using the Akaike information criterion (AIC), which trades-off goodness-of-fit with model complexity. Since AIC values may be unfamiliar, they are converted into model probabilities, which sum to one for all of the models under consideration and can be (loosely) interpreted as the probability that a model is the best (higher probabilities are better). Such model comparison methods are discussed in detail by Burnham and Anderson [31,32]. P-values are also examined to see how they differ between analyses. The analyses were performed in R (version 3.1.0) [33] and the code can be found in Additional file 1.

## Availability of supporting data

The data supporting the results are included within the article and can be found in Additional file 2.

## Findings

**Figure 3:**
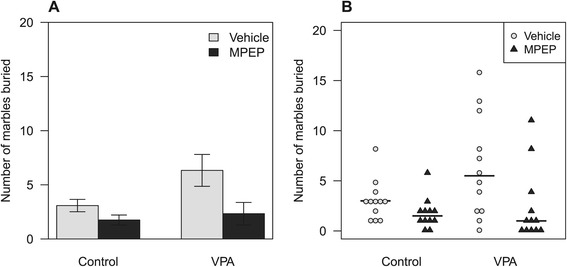
Marble burying test data.**(A)** Data from the marble burying test are displayed with a typical mean ± SEM graph, and **(B)** with all the data plotted, which highlights the skewness, unequal variances, and the discrete and bounded nature of the values. Horizontal lines are the medians.

The data in Figure 3A show the mean and the standard error of the mean (SEM) for each group. Mean and error bar graphs are common in the biomedical literature but are ill-suited for understanding the data. They obscure the discrete and bounded nature of the values, the skewness, and the unequal variances across groups. In addition, these graphs hide outliers and any unusual clusters in the data (which perhaps explains their ubiquity), and whether any animals buried all of the marbles present (censored observations). These properties are revealed by plotting the raw data as in Figure 3B. Plotting all of the data is useful for understanding the structure and relationships present, and for quality control (e.g. detecting clusters or outliers). Each point represents the number of marbles buried by one mouse, and due to the discrete nature of the data, the points are stacked beside each other, giving an impression of the shape of the distribution within each group. Even if graphs like Figure 3B are not used for publication, they should nevertheless be examined to obtain a better appreciation of the data.

**Figure 4:**
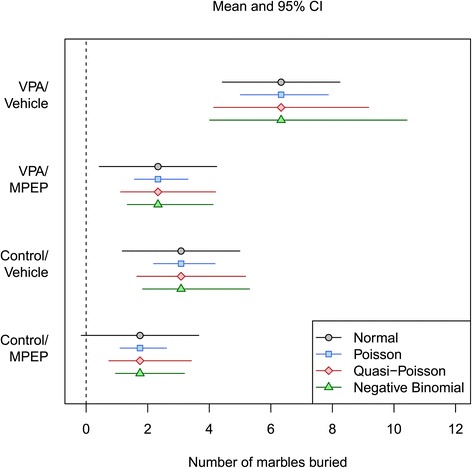
Estimated means and 95% CI for four statistical models. The normal model confidence intervals are unsuitable for this type of data as they include negative values for the Control/MPEP group. Furthermore, the intervals are of equal length (due to the homogeneity of variance assumption) and symmetric for the normal model, which does not reflect the data. The other three models have unequal and asymmetric CIs that do not include negative values.

Confidence intervals are displayed in Figure 4 for each of the four GLMs. As can be seen, the interval for the Control/MPEP group includes negative values with the normal model. In addition, the intervals are all the same width, but it was clear from Figure 3B that the equal variance assumption is not reasonable for this data (and can be confirmed with a variety of tests for homogeneity of variance: Fligner-Killeen: $\chi ^{2}_{(3)} = 10.5$, *p*=0.015; Bartlett: $K^{2}_{(3)} = 16.9$, *p*<0.001; and Levene: *F*_(3,44)_=3.78, *p*=0.017). The other three models have intervals of different sizes, they do not contain negative values, and they are asymmetric, which reflects the skewed nature of the data. The intervals for the Poisson model are too narrow because the overdispersion was not taken into account. The quasi-Poisson and negative binomial models appear sensible. Many published studies have 95% CI that include negative values (if the error bars represent SEM, approximately doubling their length gives a 95% CI) [34-38].

**Figure 5:**
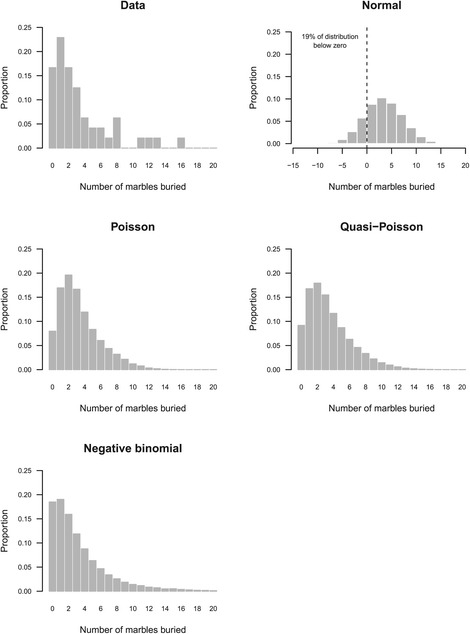
Examining model predictions. Predicted values from the four models can be compared to the data distribution to assess their suitability. The normal model is clearly inappropriate as 19% of the values are negative and the shape of the distribution looks nothing like the data. The Poisson model is better because only positive values are predicted, but it underpredicts the number of zeros (predicted = 8%, actual = 17%) and one counts, and slightly overpredicts counts between two and seven. The quasi-Poisson model is similar to the Poisson, and the distribution from the negative binomial model is the closest to the actual data.

Another way to examine the suitability of the models is with predictive simulations followed by visual checks. The idea behind predictive simulation is that if a model is appropriate and fits the data well, then new data simulated from the model should look similar to the actual data, and obvious discrepancies between actual and simulated data indicate problems with the model. The results of the predictive simulations are shown in Figure 5, and it is clear that a normal distribution is not sensible for these data as the shape of the simulated distribution looks nothing like the actual data. Furthermore, 19% of the distribution is below zero and thus contains impossible values. The Poisson distribution is much better because all values are greater than or equal to zero, and the shape is similar to the actual data distribution. Upon closer examination however it can be seen that the Poisson has too few zeros and ones. For example, the Poisson model predicts that only 8% of the data will have a value of zero, but the actual percentage is 17%. The quasi-Poisson distribution looks very similar to the Poisson, and it is difficult to determine whether it is better by visual inspection. All of the simulated Poisson values are below 20 (the number of marbles present), but a very small proportion (0.00208%) were greater than 20 with the quasi-Poisson model. The negative binomial distribution follows the actual data distribution the closest, but 0.6% of values from the negative binomial distribution were greater than 20. As mentioned previously, models for count data do not have an upper limit and they may predict values greater than the number of marbles used in the experiment. The probability of obtaining such values was very low and therefore negligible, especially compared with the 19% of impossible values from the normal model. Nevertheless, in other data sets this proportion might be much higher, in which case the count models may not be suitable and the data might be better analysed as the proportion of marbles buried using a binomial GLM. Note that the *x*-axes in Figure 5 range from 0–20 for all graphs (except for the normal model) so that direct visual comparisons can be made.

The above graphical comparisons can indicate gross deviations between actual and predicted data but are informal methods that cannot be used to discriminate between similar distributions. Quantitative methods are available to assist with choosing an appropriate model and one approach is to use a goodness-of-fit test to calculate how well a model fits the data (Table 1). A small p-value indicates a poor fit. The normal, Poisson, and quasi-Poisson models all fit the data poorly (*p*<0.0001) whereas the negative binomial model is much better (*p*=0.233). Another approach is to use information theoretic methods such as the AIC to compare models to each other, which does not provide an absolute measure of model fit, but only a relative comparison amongst the models. The more complex a model, the better it will fit a given data set; unlike the GOF tests, the AIC penalises more complex models, and thus trades-off complexity and fit. A lower AIC value is better, and the actual value has no meaning—it is only used comparatively. The negative binomial model has the lowest AIC (Table 1) and is therefore the best. The difference in AICs between the negative binomial and Poisson model (the next best model) is 33, indicating that the negative binomial is a substantial improvement over the Poisson. Converting AIC values into model probabilities shows that with high probability, the negative binomial model is the best. It is not possible to calculate an AIC for the quasi-Poisson model (a quasi-AIC measure has been developed [31], pp. 67–70 but it cannot be directly compared with the other AIC values and so is not displayed). The GOF for the quasi-Poisson is also identical to that of the Poisson and so it is not possible to discriminate between these two models based on their GOF. The quasi-Poisson is therefore difficult to compare with the other models and for that reason the negative binomial might be considered a better way to deal with overdispersion. Ver Hoef and Boveng provide a good discussion on using quasi-Poisson versus negative binomial models to account for overdispersion [39]. Based on the model predictions, the GOF statistics, and AIC values, the negative binomial model is preferred, and the normal model—the most commonly used in the literature—is the worst. These results do not imply that a negative binomial model will be the best for other data sets; the best model will need to be determined in each case.

**Table 1:** **Numeric results for different models**

**Analysis**	**VPA**	**MPEP**	**VPA × MPEP**	**GOF**	**AIC**	***P***_***model***_
Normal	0.055	**0.009**	0.178	**<0.001**	259	<0.001
Poisson	**<0.001**	**<0.001**	0.223	**<0.001**	256	<0.001
Quasi-Poisson	**0.045**	**0.006**	0.490	**<0.001**	NA	NA
Negative binomial	0.075	**0.009**	0.470	0.233	**223**	**1.000**
Wilcoxon						
	All: 0.602	All: **0.002**	NA	NA	NA	NA
	MPEP: 0.494	VPA: **0.021**	NA	NA	NA	NA
	Vehicle: 0.138	Control: **0.044**	NA	NA	NA	NA

P-values for main and interaction effects are displayed. Goodness-of-fit p-values (a small p-value indicates a poor fit), AIC values (lower is better), and model probabilities (higher is better) indicate that the negative binomial model is preferred. The nonparametric Wilcoxon tests give much larger p-values overall.

The p-values for the different analyses are listed in Table 1. The first thing to note is the p-values for the nonparametric Wilcoxon test are much larger than for the parametric methods. Also, it was not possible to test for an interaction between VPA and MPEP. The Wilcoxon test was also absent from previous comparisons because it is not possible to simulate data sets or calculate a GOF or AIC, which highlights the shortcomings of nonparametric methods. The p-values from the normal model were similar to the preferred negative binomial model (Table 1), but this will not always be the case, particularly when there are many low counts. All p-values for the effect of VPA in Table 1 are inappropriately small because the sample size was taken to be the number of animals rather than the number of pregnant females, or equivalently, the number of litters [24,28]. The correct sample size can be disregarded when performing a purely statistical comparison of methods, but in order to obtain the correct biological interpretation, a generalised linear mixed effects model was used. This is similar to the Poisson model but correctly accounts for the split-unit nature of the design. While animals in the VPA group buried 1.6 more marbles than animals in the control group, there was no strong evidence for an effect of VPA (*p*=0.192). The power to detect this effect is low since the (correct) sample size is now only 14. In addition, there were nearly twice as many VPA as control litters, which is less powerful than having an equal number of litters in the two conditions. MPEP reduced the number of marbles buried by 4.1 (*p*=0.002), and this effect was similar within the VPA and control groups (little evidence for an interaction effect: *p*=0.203). Another finding from this analysis is that the effect of MPEP varied across litters. On average, animals in the MPEP condition buried 4.1 fewer marbles, but this ranged from 0 to 10 marbles within the different litters. The variation of MPEP’s effect within litters is larger than expected by chance (*p*<0.001), assuming that the average effect of 4.1 is constant across litters, and any deviation from this value represents sampling variation. The cause of this variation is unclear.

One may argue that effects are often large and the differences between using a normal, Poisson, or negative binomial model will be small, and the correct overall conclusion will still be reached. It is true that when large effects are present, the correct qualitative conclusion can be obtained (i.e. reject or do not reject the null hypothesis) no matter how inappropriate the analysis. However, if only qualitative results are of interest then a statistical analysis can be dispensed with altogether. The purpose of an analysis is to obtain unbiased estimates of effects and an appropriate measure of uncertainty in those estimates. Models for count data have been developed, are simple to implement and interpret, and available in most statistical packages. These should be the default methods for marble burying data with normal linear models used only if they are justified.

## Conclusions

Given that the number of marbles buried are counts, statistical methods that assume normality, equal variances, and unbounded values will often be inappropriate. If a formal statistical analysis is to be conducted, then appropriate methods should be used. Furthermore, the number of marbles used and the time available to bury them should be optimised such that no (or very few) animals bury all of the marbles. This will ensure that censored observations do not bias the estimates and reduce the sensitivity of the assay. These points also generalise to other outcomes that are counts. The American National Institutes of Health (NIH) has recently recognised the need to improve the statistical skills of biologists [40], and expanding one’s repertoire of statistical methods beyond the normal linear model is a useful and straightforward step in that direction.

## Additional files

Additional file 1
**R code.** Code for the analyses is given as a plain text file.

Additional file 2
**Raw data.** Raw data from Mehta et al. [18], including the number of marbles buried, sex, and litter. Details can be found in the original publication.

## References

[1] Witkin JM (2008). Animal models of obsessive-compulsive disorder. Curr Protoc Neurosci.

[2] Jimenez-Gomez C, Osentoski A, Woods JH (2011). Pharmacological evaluation of the adequacy of marble burying as an animal model of compulsion and/or anxiety. Behav Pharmacol.

[3] Albelda N, Joel D (2012). Animal models of obsessive-compulsive disorder: exploring pharmacology and neural substrates. Neurosci Biobehav Rev..

[4] Gyertyan I (1995). Analysis of the marble burying response: marbles serve to measure digging rather than evoke burying. Behav Pharmacol..

[5] Deacon RMJ (2006). Digging and marble burying in mice: simple methods for in vivo identification of biological impacts. Nat Protoc..

[6] Thomas A, Burant A, Bui N, Graham D, Yuva-Paylor LA, Paylor R (2009). Marble burying reflects a repetitive and perseverative behavior more than novelty-induced anxiety. Psychopharmacology (Berl).

[7] Hayashi E, Kuratani K, Kinoshita M, Hara H (2010). Pharmacologically distinctive behaviors other than burying marbles during the marble burying test in mice. Pharmacology.

[8] Sugimoto Y, Tagawa N, Kobayashi Y, Hotta Y, Yamada J (2007). Effects of the serotonin and noradrenaline reuptake inhibitor (SNRI) milnacipran on marble burying behavior in mice. Biol Pharm Bull..

[9] Santini E, Huynh TN, MacAskill AF, Carter AG, Pierre P, Ruggero D (2013). Exaggerated translation causes synaptic and behavioural aberrations associated with autism. Nature.

[10] O’Hara RB, Kotze DJ (2010). Do not log-transform count data. Methods Ecol Evol..

[11] Nieuwenhuis S, Forstmann BU, Wagenmakers E-J (2011). Erroneous analyses of interactions in neuroscience: a problem of significance. Nat Neurosci..

[12] Nelder JA, Wedderburn RWM (1972). Generalized linear models. J R Stat Soc Ser A.

[13] Crawley MJ (2002). Statistical Computing: An Introduction to Data Analysis Using S-Plus.

[14] Crawley MJ (2007). The R Book.

[15] Faraway JJ (2006). Extending the Linear Model with R: Generalized Linear, Mixed Effects and Nonparametric Regression Models.

[16] Hilbe JM (2014). Modeling Count Data.

[17] Hilbe JM (2011). Negative Binomial Regression.

[18] Mehta MV, Gandal MJ, Siegel SJ (2011). mGluR5-antagonist mediated reversal of elevated stereotyped, repetitive behaviors in the VPA model of autism. PLoS One.

[19] Casella G (2008). Statistical Design.

[20] Mead R, Gilmour SG, Mead A (2012). Statistical Principles for the Design of Experiments: Applications to Real Experiments.

[21] Haseman JK, Hogan MD (1975). Selection of the experimental unit in teratology studies. Teratology.

[22] Holson RR, Pearce B (1992). Principles and pitfalls in the analysis of prenatal treatment effects in multiparous species. Neurotoxicol Teratol..

[23] Zorrilla EP (1997). Multiparous species present problems (and possibilities) to developmentalists. Dev Psychobiol..

[24] Lazic SE, Essioux L (2013). Improving basic and translational science by accounting for litter-to-litter variation in animal models. BMC Neurosci..

[25] Bolker BM, Brooks ME, Clark CJ, Geange SW, Poulsen JR, Stevens MHH (2009). Generalized linear mixed models: a practical guide for ecology and evolution. Trends Ecol Evol..

[26] Giovanoli S, Engler H, Engler A, Richetto J, Voget M, Willi R (2013). Stress in puberty unmasks latent neuropathological consequences of prenatal immune activation in mice. Science.

[27] Dias BG, Ressler KJ (2014). Parental olfactory experience influences behavior and neural structure in subsequent generations. Nat Neurosci..

[28] Lazic SE (2013). Comment on “stress in puberty unmasks latent neuropathological consequences of prenatal immune activation in mice”. Science.

[29] Zuur AF, Ieno EN, Walker NJ, Saveliev AA, Smith GM (2009). Mixed Effects Models and Extensions in Ecology with R.

[30] Gelman A, Hill J (2007). Data Analysis Using Regression and Multilevel/Hierarchical Models.

[31] Burnham KP, Anderson DR (2002). Model Selection and Multimodel Inference: A Practical Information-Theoretic Approach.

[32] Anderson DR (2008). Model Based Inference in the Life Sciences: A Primer on Evidence.

[33] Ihaka R, Gentleman R (1996). R: a language for data analysis and graphics. J Comput Graph Stat..

[34] Dagyte G, Crescente I, Postema F, Seguin L, Gabriel C, Mocaer E (2011). Agomelatine reverses the decrease in hippocampal cell survival induced by chronic mild stress. Behav Brain Res..

[35] Kim T-K, Han H-E, Kim H, Lee J-E, Choi D, Park WJ (2012). Expression of the plant viral protease nia in the brain of a mouse model of Alzheimer’s disease mitigates a-beta pathology and improves cognitive function. Exp Mol Med..

[36] de Almeida AAC, de Carvalho RBF, Silva OA, de Sousa DP, de Freitas RM (2014). Potential antioxidant and anxiolytic effects of (+)-limonene epoxide in mice after marble-burying test. Pharmacol Biochem Behav..

[37] Lugo JN, Smith GD, Arbuckle EP, White J, Holley AJ, Floruta CM (2014). Deletion of PTEN produces autism-like behavioral deficits and alterations in synaptic proteins. Front Mol Neurosci..

[38] Jury NJ, McCormick BA, Horseman ND, Benoit SC, Gregerson KA (2015). Enhanced responsiveness to selective serotonin reuptake inhibitors during lactation. PLoS One.

[39] Ver Hoef JM, Boveng PL (2007). Quasi-poisson vs. negative binomial regression: how should we model overdispersed count data?. Ecology.

[40] Collins FS, Tabak LA (2014). Policy: NIH plans to enhance reproducibility. Nature.

[41] Lazic SE (2010). The problem of pseudoreplication in neuroscientific studies: is it affecting your analysis?. BMC Neurosci..

